# Chloroform

**Published:** 1848-02

**Authors:** 


*Chloroform.*—The new anæsthetic agent, entitled as above, seems to have displaced summarily and entirely the vapor of sulphuric ether. Our readers will find in our Eclectic Department of this number, an account of its chemical nature, and the mode of preparing it, from the pen of Dr. Simpson, of Edinburg, the discoverer.

It has a great advantage over the ether in not possessing irritating properties, but, on the other hand, being fragrant and agreeable. On this account it is respired with ease and comfort, and may be employed in cases in which, owing to irritability of the pulmonary apparatus, the vapor of ether cannot be inhaled. Owing to the ease with which it may be respired no inhaling machine is required, but a concave sponge or handkerchief saturated with the fluid, and applied to the mouth suffices for all practical purposes. It remains to determine by comparative observations which of the two agents exerts the most satisfactory results, or which is less objectionable than the other.

